# Physicochemical Properties, Rheological Characteristics, Flavor Profile and Antioxidant Activity of Fermented Plant-Based Alternative to Yoghurt from *Avena sativa* L. and *Prunus dulcis* (Mill.) D. A. Webb

**DOI:** 10.3390/foods15091529

**Published:** 2026-04-28

**Authors:** Menghan Ma, Mengjie Li, Duo Feng, Jing Wang

**Affiliations:** 1 College of Food Science and Engineering, Qingdao Agricultural University, Qingdao 266000, China; mmh0725@163.com; 2 Institute of Food and Nutrition Development, Ministry of Agriculture and Rural Affairs, Beijing 100081, China; limj0804@126.com (M.L.); 15525926785@163.com (D.F.)

**Keywords:** oats, almonds, plant-based yoghurt, volatile flavor compounds, antioxidant activity

## Abstract

This study compared oat yoghurt (OY), almond yoghurt (AY), oat–almond yoghurt (OAY), and an unfermented oat–almond milk (OAM) to clarify how blending and lactic fermentation affect fermented plant-based alternatives to yoghurt. Nutritionally, OAY showed a balanced profile (protein 2.87 g/100 g; fat 5.18 g/100 g), intermediate between AY (3.29 g/100 g, 8.89 g/100 g) and OY (2.39 g/100 g, 3.30 g/100 g). Fermentation enhanced physical stability, as OAY showed higher water-holding capacity (58.08%) and high viscosity (5381.49 mPa·s), together with the highest viable lactic acid bacteria count (7.1 log10 CFU/g). Scanning electron microscopy revealed that co-fermentation formed a denser, more cohesive multiphase gel network with reduced pore size compared with OAM and AY. All samples exhibited shear-thinning behavior; dynamic rheology indicated weak gel features (G′ > G″), and OAY showed the highest G′, implying a reinforced network likely associated with interactions between oat β-glucan and almond proteins during fermentation. Volatile profiling by GC–MS identified 117 compounds, and OAY exhibited the greatest total volatiles (523.02 μg/kg), exceeding OY (397.43 μg/kg) and OAM (195.73 μg/kg), indicating improved aroma complexity and consumer acceptability. In conclusion, our study will provide quantifiable formulations for the development of highly acceptable oat and almond-based plant-based yoghurt. Most importantly, it also offers additional dairy products for individuals with gluten allergies and lactose intolerance.

## 1. Introduction

Milk allergy ranks among the most prevalent food allergies in infancy and early childhood [1], with multiple studies indicating that the majority of affected children may outgrow this allergic reaction as they age [2]. Conversely, lactose intolerance affects approximately 65% of the global population, being particularly prevalent in East Asia [3]. Consequently, plant-based dairy alternatives are gaining increasing popularity among consumers due to their sustainable production characteristics. The primary motivations for choosing plant-based alternatives over traditional milk stem from concerns regarding animal welfare, environmental protection, and health considerations. Plant-based yoghurts are produced by fermenting plant-based suspensions or aqueous extracts using specific microbial culture [4]. It primarily utilizes plant-based products with high protein content as raw materials, typically sourced from legumes, nuts, and grains [5]. Compared to traditional animal-derived fermented yoghurts, plant-based yoghurts have emerged as a developmental trend within dairy products due to their broader dietary adaptability, richer variety of ingredients, and cholesterol-free profile [6]. Nevertheless, due to the compositional complexity of plant proteins and polysaccharides and their distinct buffering and gelation behaviors, plant-based yoghurts frequently suffer from weak gel networks, pronounced syneresis [7], coarse mouthfeel, and undesirable “raw/green” off-notes [8]. These technological and sensory limitations remain key barriers to broader consumer acceptance and industrial application.

Oats (*Avena sativa* L.) and almonds (*Prunus dulcis* (Mill.) D. A. Webb.), as nutrient-rich plant-based raw materials, hold significant potential in the development of health foods. Oats are abundant in β-glucans and dietary fiber, with their β-glucans shown to aid in regulating blood sugar, lowering cholesterol, and improving gut health [9]. Oat protein has a well-balanced amino acid profile, with a higher lysine content than conventional cereals, which can compensate for the lysine deficiency in proteins from other cereal grains [10]. Almonds are renowned for their high content of unsaturated fatty acids and high-quality protein. Studies have shown that almonds have a protein content ranging from 20% to 25%, making them a high-protein plant-based food [11]. However, almond protein exhibits relatively low lysine content [12], necessitating combination with other proteins to optimize amino acid balance. Both raw materials are plant-based materials, typical representatives of coarse grains and nuts, enabling nutritional complementarity. Furthermore, both are suitable for lactic acid bacteria fermentation [13], rendering them excellent raw materials for plant-based fermentation. Fermentation enhances the digestibility and bioavailability of nutrients while generating or releasing bioactive compounds [14]. Although quality changes in oat- or nut-based fermented matrices have been reported [15,16], systematic evidence is still limited regarding how oat–almond co-fermentation shapes product quality across multiple scales, especially when benchmarked against both unfermented systems and single-ingredient fermented counterparts.

Therefore, this research compared oat yoghurt (OY), almond yoghurt (AY), oat–almond yoghurt (OAY), and an unfermented oat–almond milk (OAM) to elucidate the effects of ingredient combination and lactic fermentation on the quality of plant-based yoghurt alternatives. This research analyzed the structural assembly, rheological properties, and volatile composition of the mixed system in comparison to its individual components and an unfermented counterpart, this research seeks to clarify the mechanisms underlying quality improvement in composite fermented plant-based alternatives to yoghurt, thereby facilitating the development of products suitable for consumers with specific dietary requirements.

## 2. Materials and Methods

### 2.1. Materials and Reagents

The raw materials used for the preparation of the yoghurt substitutes included oats, almonds, and sucrose. Oats were purchased from October Rice Field Group Co., Ltd. (Shenyang, China), and almonds were obtained from Maoming Baifude Ecological Food Co., Ltd. (Maoming, China). Sucrose was sourced from COFCO Group Co., Ltd. (Beijing, China).

For fermentation, a commercial direct-vat-set starter culture (Type YO-MIX725) was employed, which was purchased from Danisco (China) Co., Ltd. (Shanghai, China). This starter culture consisted of a mixed strain formulation containing *Streptococcus salivarius* subsp. *thermophilus*, *Lactobacillus acidophilus*, *Lactobacillus delbrueckii* subsp. *bulgaricus*, and *Bifidobacterium animalis* subsp. *lactis*.

Chemical reagents used in the analyses were of analytical grade. Na_2_CO_3_, NaOH, cyclohexanone, and methanol were purchased from China National Pharmaceutical Group Chemical Reagents Co., Ltd. (Shanghai, China). For microbiological analysis, MRS agar was obtained from Beijing Luqiao Technology Co., Ltd. (Beijing, China). Folin–Ciocalteu reagent was sourced from Beijing Solarbio Science & Technology Co., Ltd. (Beijing, China), and gallic acid was provided by Tianjin Guangfu Fine Chemical Research Institute (Tianjin, China).

### 2.2. Preparation of Fermented Plant-Based Alternative to Yoghurts

The operational steps for fermenting a plant-based alternative to yoghurt were referenced from Wang [17] with minor modifications. OAY, OY and AY were produced using the same processing procedure, differing only in raw material composition. For OAY, roasted and peeled almonds were mixed with roasted oats at a mass ratio of 4:6, whereas OY and AY were prepared using roasted oats and roasted almonds as the sole raw material, respectively.

All raw materials were blended with distilled water at a solid/liquid ratio of 1:10 (g:mL, dry weight basis) using a high-speed blender (PBJ-09, Meihua Life Electric Appliance Co., Ltd., Chengdu, China). The resulting slurry was filtered through an 80-mesh sieve and homogenized at 25 MPa for three cycles using a high-pressure homogenizer (WH-1500, Suzhou Antos Nano Technology Co., Ltd., Suzhou, China). Sucrose (6%, *w*/*v*) was then added and fully dissolved. The mixtures were pasteurized at 95 °C for 20 min and subsequently cooled to the inoculation temperature. A commercial direct-vat-set starter culture (0.1%, *w*/*v*) was added, followed by fermentation at 40 °C for 8 h. After fermentation, all yoghurt samples were stored at 4 °C until further analysis.

**Figure 1:**
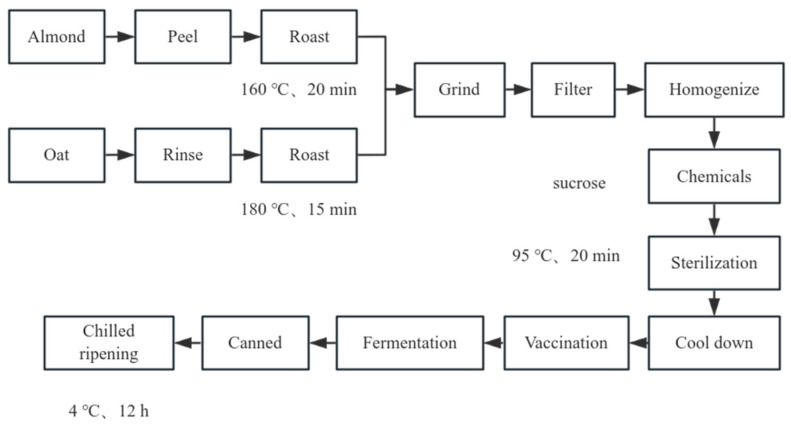
Fermented plant-based alternative to yoghurt process flow diagram.

The preparation of oat almond milk (OAM) was referenced from Zhai et al. [18] with minor modifications. Roasted and peeled almonds were mixed with roasted oats at a mass ratio of 4:6 and processed following the same blending, filtration, homogenization (25 MPa, three cycles), sucrose addition (6%, *w*/*v*), and pasteurization conditions as described for the yoghurt samples. After pasteurization, the mixture was cooled and stored at 4 °C without fermentation. The specific operational procedure is shown in Figure 1.

### 2.3. Nutritional Information

Our research refers to the method described by Oğuz [19]: the protein content of yoghurt was determined by the Kjeldahl method, and the fat content was measured using the Soxhlet extraction method. The carbohydrate content was estimated using the difference method. Total solids content (TSC) was determined according to Obaroakpo [20]: 50 g of yoghurt sample was placed in a constant temperature oven (model T535, Beideng Jinghui Technology Co., Ltd., Shenzhen, China) and dried in an oven at 100 ± 5 °C for 2 h, then cooled in a desiccator for 0.5 h before final weighing. The calculation formula is as follows:

(1) $$TSC(\%)=\frac{m_{1}-m_{2}}{m_{1}-m_{3}}\times 100$$

m_1_ denotes the total mass of the beaker and sample (g);
m_2_ denotes the total mass of the beaker and sample after drying to constant weight (g);
m_3_ denotes the mass of the beaker after drying to constant weight (g).

### 2.4. Physicochemical Analysis

pH was determined using a digital pH meter (model pH-100, Shanghai Lichen Instrument Technology Co., Ltd., Shanghai, China). Total titratable acidity (TTA) was measured as follows: 10 g of sample was homogenously mixed with 90 mL of distilled water, then titrated with 0.1 mol/L NaOH solution until the endpoint pH of 8.3 was reached. The volume (mL) of titrant consumed during this process served as the quantitative unit for acidity. The determination method for water-holding capacity (WHC) was modified from the approach described by Wang et al. [21]. Specifically, 10 g of yoghurt sample was weighed into a centrifuge tube and centrifuged at 4 °C for 30 min at 4000 rpm. The supernatant was discarded, and the residual sample weight and total weight of the centrifuge tube were recorded. Viscosity was measured using a viscometer (model NDJ-5S8S, Shanghai Lichen Instrument Technology Co., Ltd., Shanghai, China) at a rotational speed of 60 rpm.

### 2.5. Microbiological Analysis

The total lactic acid bacteria count (TLC) for the four sample groups was enumerated on MRS agar. Diluted samples were evenly spread onto plates and incubated at 37 °C for 48 h. The TLC was expressed as colony-forming units per gram of yoghurt (CFU/g) [22].

### 2.6. Texture Analysis

The texture analyzer (model Universal TA, Shanghai Tengbo Instrument Technology Co., Ltd., Shanghai, China) employed a flat-bottomed cylindrical probe (SMS P/75). Test parameters were as follows: pre-test speed 2.0 mm/s, test speed 2.0 mm/s, post-test speed 2.0 mm/s, cycle count 2, trigger force 5 g, and compression interval pause time 5 s. Each sample was measured three times, with the mean value recorded [23]. Hardness, adhesiveness, chewiness, resilience, and cohesiveness were measured for four sets of samples.

### 2.7. Microstructure

Four sets of yoghurt samples preserved at 4 °C were uniformly thinly coated onto the inner walls of Petri dishes. After freezing in liquid nitrogen, they were promptly placed in a vacuum freeze-dryer for desiccation (model LGJ-10, Beijing Qihang Technology Co., Ltd., Beijing, China). Subsequently, gold coating was applied via ion sputtering. The samples were then prepared for scanning electron microscopy (SEM) (model SU8100, manufactured by Hitachi High-Tech Corporation, Tokyo, Japan) observation. Finally, the microstructure of the yoghurt samples was photographed and examined at an acceleration voltage of 10 kV and a magnification of 6000× [24].

### 2.8. Rheological Properties

The rheometer (model HAAKE Mars40, Thermo Fisher Scientific, Suzhou, China) was equipped with a stainless steel flat plate system with a diameter of 40 mm, a plate spacing of 1000 μm, and a measurement temperature of 4 °C [25]. Viscosity characteristics were measured using a flow sweep program, with shear rates ranging from 0.01 to 100 s^−1^ (logarithmically increasing). Dynamic viscoelasticity was measured using a frequency sweep program, with frequencies ranging from 0.1 to 100 Hz and a strain value set at 2% (within the linear viscoelastic range). The results for the storage modulus (G′) and loss modulus (G″) were plotted on logarithmic–logarithmic coordinates.

### 2.9. Electronic Nose and GC-MS

The electronic nose method (model PEN3, AIRSENSE GmbH, Schwerin, Germany) was modified based on the method described by Wu [26]. A 5 mL sample was placed in a 20 mL headspace vial and incubated at 50 °C in a water bath for 30 min, after which volatile flavor compounds were determined using the electronic nose. The GC-MS analysis (model TSQ8000, Thermo Fisher Scientific, Suzhou, China) was modified according to the method of Huang Kai [22]. 5 mL of yoghurt sample was weighed and 2 µL of internal standard (cyclohexanone) added. The mixture was placed in a 50 mL solid-phase microextraction (SPME) vial. A manual syringe fitted with a 2 cm, 50/30 µm DVB/CAR/PDMS Stable Flex fiber was inserted. The sample was thermostated at 60 °C on a magnetic stirrer (stirring speed 150 rpm) for 50 min of headspace extraction. Upon completion, the fiber was immediately withdrawn and inserted into the gas chromatograph inlet (temperature 250 °C) for 6 min of thermal desorption prior to injection.

### 2.10. Preparation of Yoghurt Extract

Place a 10 g yoghurt sample in a 50 mL centrifuge tube and add 15 mL of acidified methanol. Homogenize the mixture at 12,000 rpm for 1 min, then refrigerate at −20 °C for 1 h to ensure complete protein precipitation. Subsequently, centrifuge at 7000 rpm for 10 min at 4 °C. Filter the supernatant through a 0.22 µm membrane for subsequent determination of polyphenol content and antioxidant activity [27].

#### 2.10.1. Total Phenolic Content

Total phenolic content (TPC) was performed with slight modifications to the method described by Zuo [28]: A total of 1 mL of diluted yoghurt extract was mixed with 2 mL of Folin–Ciocalteu reagent. After thorough mixing, the reaction was allowed to proceed for 6 min. Subsequently, 2 mL of 10% Na_2_CO_3_ solution was added, and the mixture reacted at room temperature for 60 min. Absorbance was measured at a wavelength of 765 nm. A standard calibration curve was generated using a series of gallic acid solutions (4, 8, 12, 24, 32, 40 μg/mL). Total phenolic compounds were estimated based on the gallic acid calibration curve, with the results expressed as gallic acid equivalents (TPC, mg GAE/100 g).

#### 2.10.2. DPPH Radical Scavenging Activity

This was based on the method of Bolek [29], with minor modifications. Briefly, 2 mL of DPPH solution was mixed with 2 mL of sample extract. Following thorough mixing, the reaction mixture was incubated in darkness for 30 min. Absorbance (A_a_) was subsequently measured at 517 nm. The control group (A_b_) employed 2 mL of absolute ethanol in place of the sample extract. The DPPH radical scavenging rate was calculated using the following formula:

(2) $$DPPH\;Radical\;Scavenging\;Rate\;(\%)=\frac{A_{b}-A_{a}}{A_{b}}\times 100$$

#### 2.10.3. ABTS Radical Scavenging Activity

This was based on Aljewicz’s method with minor modifications [13]. Briefly, 2 mL of ABTS working solution was added to a test tube, followed by 1 mL of sample extract. The mixture was shaken to ensure thorough mixing and allowed to react for 10 min at room temperature in the dark. The absorbance (A_a_) was measured at 734 nm. For the control group (A_b_), 1 mL of anhydrous ethanol was used in place of the sample solution. The formula for calculating the ABTS radical scavenging rate is as follows:

(3) $$ABTS\;Radical\;Scavenging\;Rate\;(\%)=\frac{A_{b}-A_{a}}{A_{b}}\times 100$$

#### 2.10.4. Hydroxyl Radical Scavenging Activity

We referred to the method of Wang [30] with minor modifications. In separate test tubes, sequentially add 2 mL of 9 mmol/L ferrous sulphate solution, 2 mL of 9 mmol/L salicylic acid/ethanol solution, the sample solution, and 2 mL of 8.8 mmol/L hydrogen peroxide solution. After shaking the mixture for 10 s, allow the reaction to proceed at room temperature for 1 h. Following the reaction, measure the absorbance (A_1_) at 510 nm. For the blank control (A_0_), replace the sample solution with distilled water. The hydroxyl radical scavenging rate is calculated using the following formula:

(4) $$Hydroxyl\;Radical\;Scavenging\;Rate\;(\%)=\frac{A_{0}-A_{1}}{A_{0}}\times 100$$

### 2.11. Sensory Evaluation

Ten food science students aged 22–28 with balanced gender distribution evaluated four sample groups. Water was provided during tasting breaks. The sensory attributes assessed included acidity, color, taste, odor, texture and acceptance for each sample. A five-point scale (0 = extremely dislike; 1 = dislike; 2 = moderately dislike; 3 = neither like nor dislike; 4 = moderately like; 5 = extremely like) was used to quantify sensory characteristics. The overall sensory score was calculated based on these evaluations.

### 2.12. Data Processing and Analysis

All experiments were repeated three times. The results are presented as the mean ± standard deviation. Statistical analysis was performed using IBM SPSS Statistics (version 26.0). One-way analysis of variance (ANOVA) was conducted, followed by Tukey’s post hoc test for multiple comparisons. Values were considered statistically significant at *p* < 0.05. Figures and tables were generated using Origin 2025 (OriginLab Crop., Northampton, MA, USA) and GraphPad Prism 10.1.2 (GraphPad Software, Inc., La Jolla, CA, USA).

## 3. Results and Discussion

### 3.1. Nutritional Analysis

Prior to the nutritional analysis, the manufacturing conditions were optimized to ensure product stability. As shown in Table 1, AY exhibits significantly higher protein content (3.29 g/100 g) and fat content (8.89 g/100 g) compared to the other samples, likely attributable to the inherently higher protein and fat levels inherent in almonds. OAY exhibited protein (2.87 g/100 g) and fat (5.18 g/100 g) contents intermediate between AY and OY, effectively addressing the low protein content of oat yoghurt and the high fat content of almond yoghurt. No significant differences in nutritional components were observed between OAY and OAM. Furthermore, during fermentation, fats may break down into fatty acids, resulting in OAY’s fat content being lower than that of OAM. This trend has also been observed in the production of pumpkin yoghurt [31]. The carbohydrate content of OAY (6.17 g/100 g) was slightly lower than that of OAM. This reduction in carbohydrates may be attributed to the hydrolysis of carbohydrates by amylase secreted by microorganisms [32]. Microorganisms may also utilize carbohydrates as an energy source during fermentation [33]. OY exhibits the highest total solids content (14.35 g/100 g), likely attributable to oats’ inherent high carbohydrate and soluble dietary fiber content [34].

### 3.2. Physicochemical and Microbiological Analysis

As shown in Table 2, significant differences exist in the physicochemical characteristics of the four sample groups. Research indicates that the optimal pH range for commercial fermented dairy products is 3.27 to 4.59 [18]. Both AY and OAY fell within this optimal pH range. Total acidity (TTA) exhibited a negative correlation with pH. The acidity levels of AY, OY, and OAY all met the requirement of acidity ≥ 70 °T stipulated in the Chinese standard GB 19302-2025 [35]. OY exhibited significantly higher water-holding capacity (WHC) than the other three groups, attributable to the strong hydrophilicity of oat β-glucan [36], which reduces water separation through interfacial interactions. AY demonstrated the lowest WHC, as almond protein readily flocculates at pH 4.2, forming a porous structure incapable of effective water retention [37]. Data indicate that the trend in WHC aligns with viscosity. Following fermentation, OAY exhibited higher viscosity than OAM. This suggests that acids produced during lactic acid fermentation promote plant protein gelation, thereby establishing a more robust network structure [38]. Furthermore, the total lactic acid bacteria counts in AY, OY, and OAY all exceeded the limits stipulated by the Chinese standard GB 19302-2025 [35]. Among these, the composite yoghurt OAY exhibited a higher total lactic acid bacteria count than the single-ingredient yoghurts OY and AY.

### 3.3. Texture and Microstructure

#### 3.3.1. Texture Characterization Analysis

Texture most directly reflects a product’s sensory and quality characteristics [39]. Evaluating yoghurt texture involves assessing hardness, adhesiveness, elasticity, cohesiveness, and stickiness. As shown in Table 3, OY exhibited the highest values for hardness, adhesiveness, and stickiness, yet its elasticity and cohesiveness were lower than OAY. Fermentation significantly improved the textural properties of the samples [40]. Following fermentation, OAY and OAM exhibited significant differences in hardness, adhesiveness, elasticity, and viscous texture. This occurs because acids produced by lactic acid bacteria during fermentation induce protein denaturation and cross-linking, forming a stronger gel structure that effectively encapsulates water and fat [41]. In comparison, AY exhibited the lowest values across all textural characteristics and demonstrated significant differences from the other samples. This may be attributable to the incompatibility between the starter culture and the almond-based yoghurt matrix, resulting in incomplete fermentation.

#### 3.3.2. Microstructural Analysis

**Figure 2:**
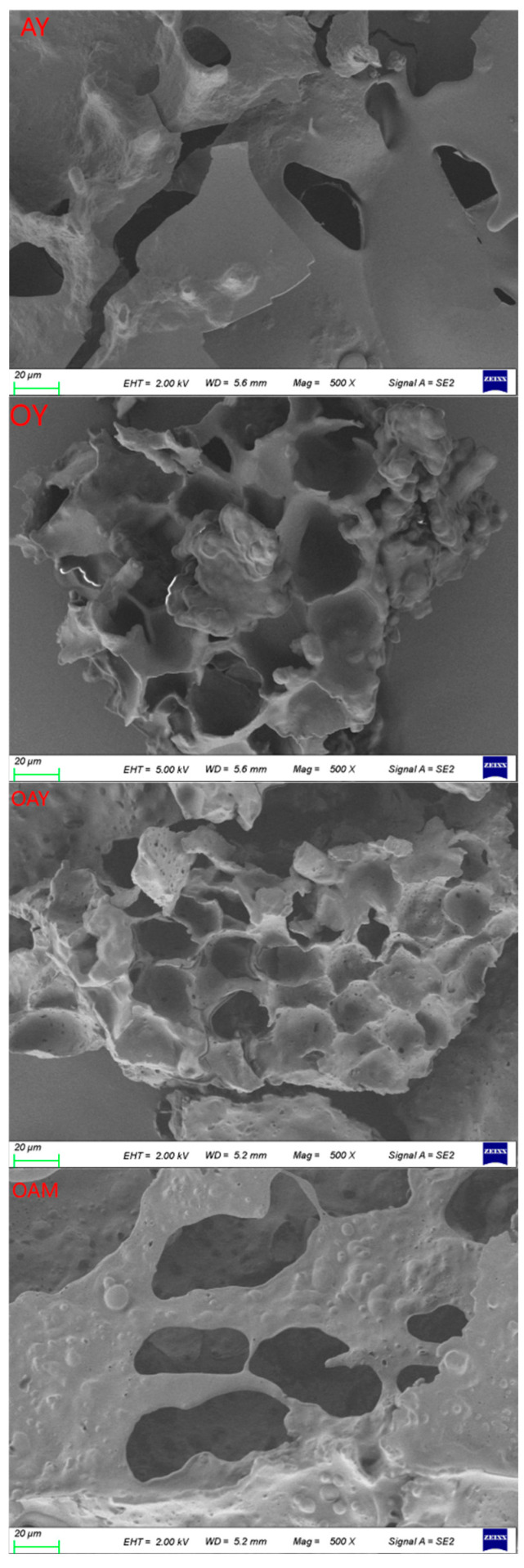
Microstructure of different yoghurt samples at 100× magnification: AY (almond yoghurt), OY (oat yoghurt), OAY (oat almond yoghurt), OAM (oat almond milk).

Changes in texture may be related to microstructure. Low-temperature scanning electron microscope images of each yoghurt sample are shown in Figure 2. Compared to OY, AY exhibits a looser, less compact structure with visible larger pores. This may be attributed to the high-viscosity dietary fibers in oats (such as β-glucan), which possess strong water-holding capacity and colloid-strengthening effects. These fibers form composite gels with the protein network, thereby creating a denser, more uniform structure [42]. Moreover, OAM exhibits a loose structure with larger pores, demonstrating low internal cohesion and a lack of gel network formation. Following fermentation, the interaction between almond protein and fat within OAY generates a more complex, multiphase gel network system. This results in a structure characterized by enhanced cohesiveness and compactness, alongside a significant reduction in pore size. This uniform, dense three-dimensional network structure facilitates the adhesion of extracellular polysaccharides produced by probiotics [43]. Extracellular polysaccharides can enhance the WHC and viscosity of yoghurt [44].

### 3.4. Rheological Behavior

**Figure 3:**
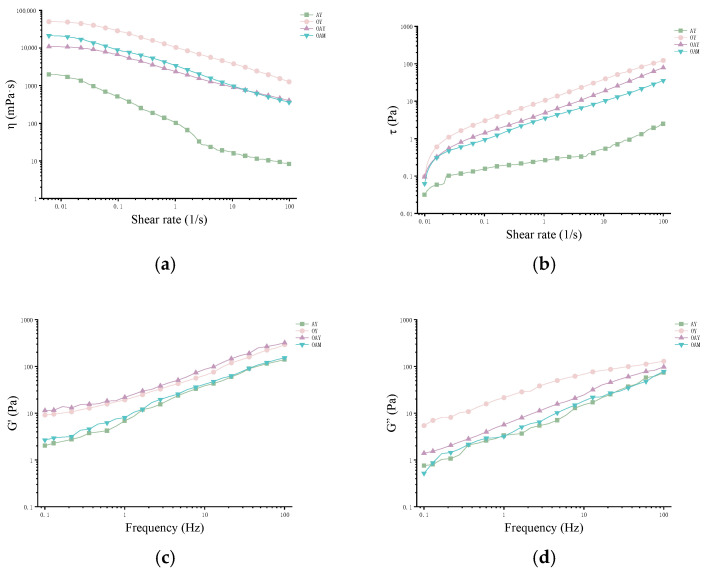
Rheological properties of different yoghurt samples: (**a**) apparent viscosity; (**b**) stress; (**c**) storage modulus; (**d**) loss modulus.

The investigation of rheological properties plays a significant role in product transportation, handling, and consumption. As shown in Figure 3, all samples exhibited shear thinning behavior across the tested shear rate range, characteristic of pseudoplastic fluids. This indicates that their microstructure undergoes reversible or partially reversible disruption and reorganization under shear stress [45]. The apparent viscosity at a shear rate of 50 s^−1^ was selected for comparing different samples, as this value is considered relevant to consumers’ perception of food viscosity [46]. The apparent viscosity of AY was slightly lower than the reported value for dairy-based yoghurt (approximately 2.3 ± 0.3 Pa·s) [47], indicating that AY has a slightly thinner consistency than dairy-based yoghurt. The apparent viscosity values of the other three samples were higher than that of dairy-based yoghurt, suggesting that these samples possess a higher viscosity. The stress/shear rate curves in Figure 3b reveal a distinct yield stress in the low shear rate region for the fermented samples. The dietary fibers present in OY and OAY, such as oat β-glucan, may enhance gel continuity through filling and physical cross-linking effects, thereby increasing yield stress and structural strength.

To further investigate the internal structure of the yoghurt, dynamic rheological parameters were measured, including the storage modulus (G′) and loss modulus (G″). As shown in Figure 3c,d, all four sample groups exhibited G′ > G″, characteristic of typical weak gel-like solid behavior [48]. With increasing frequency, both G′ and G″ exhibited a gradual upward trend, indicating that the gel network structure possesses good structural stability and frequency insensitivity. OAY exhibited the highest G′. This may stem from the potential formation of interpenetrating or phase-separated composite networks between oat β-glucan and almond protein during fermentation. The β-glucan solution phase may fill the pores of the protein gel phase, forming a continuous phase structure that significantly enhances the mechanical strength and deformation resistance of the overall network. This phenomenon has also been verified in studies of quinoa/soy yoghurt [24].

### 3.5. Analysis of Volatile Flavor Compounds

#### 3.5.1. Electronic Nose

**Figure 4:**
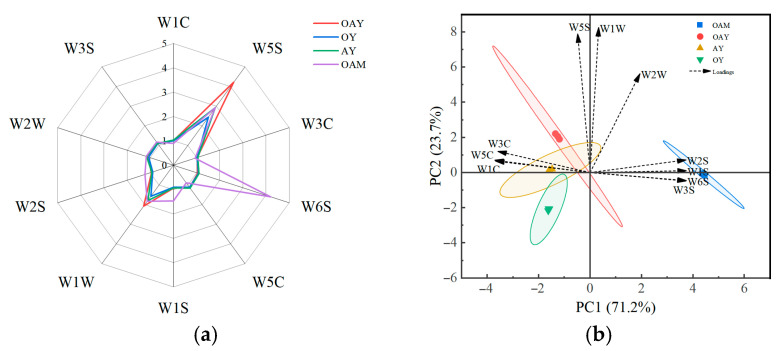
(**a**) Radar chart of sensor response intensity for different yoghurt samples; (**b**) PCA and loadings analysis of the aromas of different yoghurt samples.

Figure 4a displays a radar chart of electronic nose sensor responses for four distinct yoghurt samples. The diagram reveals that volatile compounds in all four yoghurt samples elicited responses from every sensor. The performance characteristics of the electronic nose sensor are described in Table 4. The W5S, W6S, and W1W sensors—sensitive to nitrogen oxides, hydrogen, sulfides, and terpenes—exhibited more pronounced response variations. This indicates these three sensors made significant contributions to detecting volatile compounds within the yoghurt samples.

Analysis of the contribution rates of sensors to discrimination reveals that along the PC1 axis, sensors W1C, W5C and W3C are positioned at a considerable distance from sensors W2S, W1S, W6S and W3S, distributed respectively along the positive and negative axes of PC1. This indicates that aromatic components exhibit stronger correlations with OAY, OY, and AY, while alcohols, aldehydes, ketones, alkanes, and hydrogen compounds show stronger correlations with OAM. Furthermore, principal component analysis (PCA) was conducted on the volatile compounds in different yoghurt samples. The results are presented in Figure 4b. The cumulative contribution rate of PC1 and PC2 reached 94.9%, sufficiently reflecting the sample information and indicating that PC1 and PC2 can be used to describe the primary characteristics of the aroma compounds in the samples. The OAM sample occupied a distinct, non-overlapping region in the scatter plot compared to the other samples, indicating the electronic nose’s ability to effectively differentiate fermented yoghurt from unfermented yoghurt. Although the ellipses representing OAY, OY, and AY partially overlapped, the electronic nose could not fully distinguish the flavor characteristics of these yoghurt samples. Consequently, GC-MS analysis was employed to further characterize the flavor compounds present in the samples, demonstrating significant differentiation primarily by W5S, W1W, and W2W.

#### 3.5.2. Gas Chromatography/Mass Spectrometry (GC-MS) Analysis

**Figure 5:**
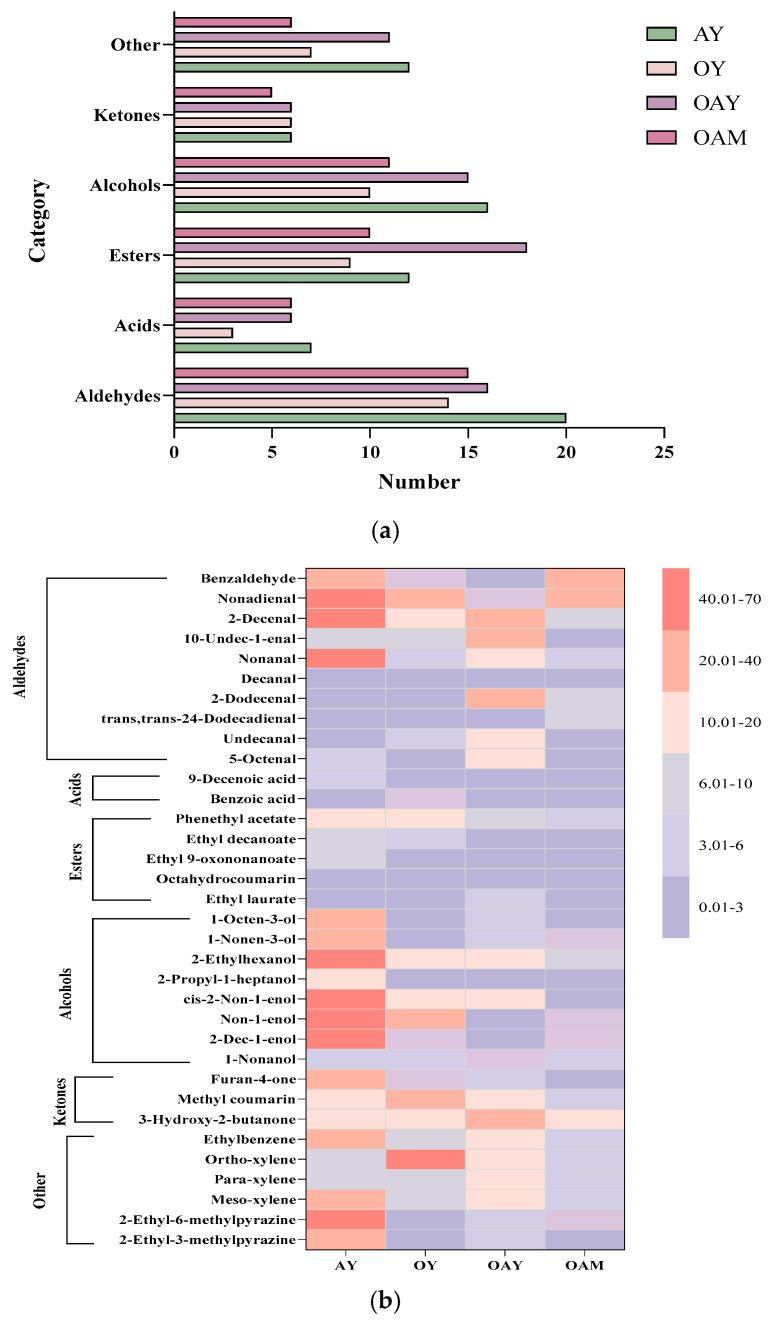
(**a**) Number of volatile flavor compounds in different yoghurt samples; (**b**) heatmap analysis of volatile compounds in different yoghurt groups.

Four sets of yoghurt samples underwent pretreatment prior to sampling and analysis. Volatile flavor compounds in five sets of yoghurt were enriched using solid-phase microextraction (SPME), followed by separation and detection via gas chromatography/mass spectrometry (GC-MS). A total of 117 volatile compounds were detected across the four yoghurt samples, encompassing aldehydes, acids, esters, ketones, alcohols, hydrocarbons, alkenes, pyrazines, and other compounds. The results are presented in Figure 5a. Researchers have discovered that the flavor profile of yoghurt is formed by the combined action of multiple flavor compounds, including alcohols, acids, aldehydes, ketones, and esters. However, not every flavor compound plays a decisive role in the sample’s taste; only those exceeding the sensory threshold can be perceived and contribute to specific flavor characteristics [49,50]. Detailed results are presented in the Appendix A. Based on this table, common volatile compounds across the four groups of yoghurt samples were summarized, and a heatmap was generated to compare their content variations, as shown in Figure 5b.

**Figure 6:**
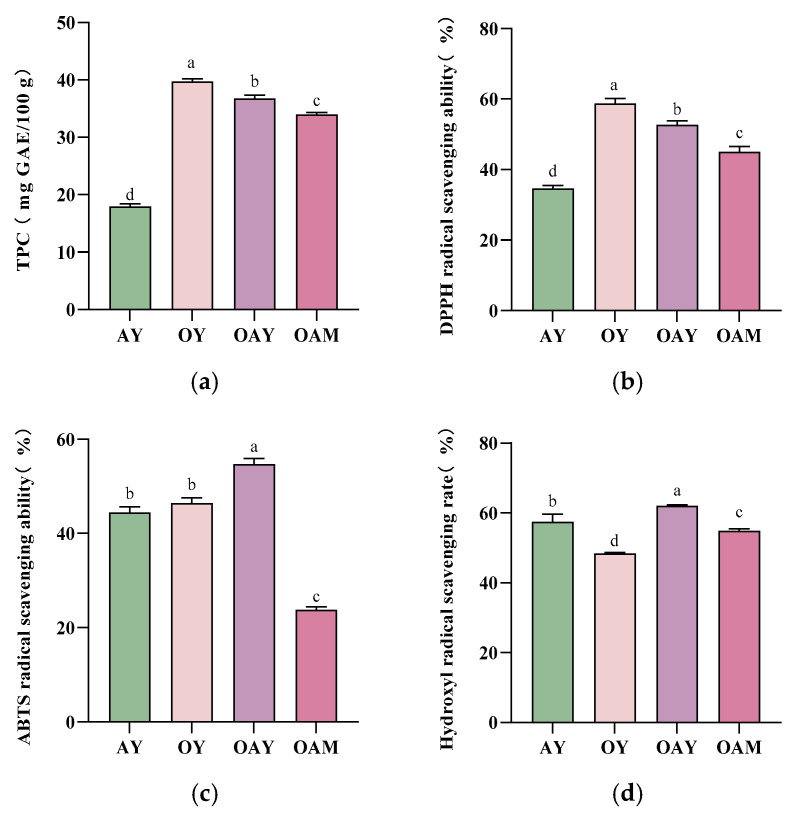
Total phenolic content and antioxidant activity of different yoghurt groups: (**a**) total phenolic content of different yoghurt samples; (**b**) DPPH radical scavenging rate; (**c**) ABTS radical scavenging rate; (**d**) hydroxyl radical scavenging rate. Identical letters in the figure indicate no significant difference (*p* < 0.05), while different letters denote significant differences (*p* < 0.05).

Aldehydes constitute key components of the characteristic flavor profile in yoghurt. They typically originate from processes such as fatty acid metabolism, amino acid transamination reactions, and Strecker degradation, while also serving as primary sources of various oxidative flavor compounds [51]. According to the Supplementary Table and data in Figure 5, 2-decene-1-aldehyde, 10-undecenal, nonanal, decanal, 2-dodecenal, 5-octenal, and undecanal were detected in all four yoghurt sample groups. Nonanal exhibits rose and citrus aromas, while 5-octenal exhibits grassy, fatty, citrus, and cantaloupe-like notes. These compounds constitute important flavor components in yoghurt. Notably, the concentration of 10-undecenal exhibited significant variation before and after fermentation in OAY (31.54 μg/kg) and OAM (1.94 μg/kg), a finding also illustrated in Figure 6. Furthermore, benzaldehyde was detected in three groups of samples without oats, while cis-7-tetradecanal was found in three groups without almonds. Compared to the other three groups, pure oat fermented yoghurt (OY) exhibited relatively lower aldehyde content. Aldehyde compounds possess low sensory thresholds and constitute an important component of yoghurt flavor compounds.

Acidic compounds, as the primary flavor components in yoghurt, are predominantly perceived through taste. 9-Dechlorobenzoic acid exhibits a fatty, waxy character with subtle fruity and milky undertones, detected in all four yoghurt categories. Benzoic acid and hexanoic acid were identified in three of the samples—excluding OY. Acidic compounds primarily result from lactic acid bacteria metabolizing sugars and amino acids. They constitute one of yoghurt’s principal flavor components, providing a refreshing palate sensation [52].

Esters constitute another crucial class of flavor compounds in yoghurt. They typically possess low detection thresholds and make a significant contribution to the overall flavor profile [53]. Among the detected ester compounds, substantial differences were observed across the four sample groups. In AY, ethyl undecenoate and ethyl phenylacetate were predominantly detected, exhibiting floral, fruity, wine-like, and whisky-like aroma characteristics. In OY, ethyl phenylacetate was the predominant compound detected. In OAY, ethyl tridecanoate and octyl phenylacetate were the main esters detected, contributing aromas dominated by sweet rose and honey, alongside subtle nuances of fruitiness and animal notes. The ester content in OAM is relatively low, which can be attributed to esters typically originating from metabolic processes dominated by lactic acid bacteria acting in concert with other microorganisms, and being regulated by fermentation conditions and post-ripening parameters [54].

A rich array of alcohol compounds was detected across the four yoghurt groups. All four groups contained 1-octen-3-ol, cis-2-nonen-1-ol, and 2-propyl-1-heptanol. 1-Octen-3-ol exhibits odors reminiscent of lavender, rose, mushroom, and hay, while cis-2-nonen-1-ol possesses green, greasy, and cucumber-like aromas. Although alcohols constitute a significant proportion of the volatile flavor compounds in yoghurt, their contribution to the overall flavor is relatively minor due to their generally higher sensory thresholds compared to other volatile compounds present in yoghurt.

Ketones are key compounds influencing yoghurt flavor, typically formed through processes such as unsaturated fatty acid oxidation, amino acid degradation, and microbial metabolism. Tetrahydropyranone, 3-hydroxy-2-butanone (acetylacetone), and 2-nonanone were detected in all four sample groups. Tetrahydropyranone has a grassy aroma; 3-hydroxy-2-butanone contributes creamy, buttery and yoghurt-like notes; and 2-nonanone exhibits a cheesy character. The number of ketone types detected showed little variation between samples: OAM contained five, while the other samples contained six.

Aromatic hydrocarbons such as toluene and ethylbenzene were detected in all four groups of yoghurts. Some of these aromatic compounds originated directly from the raw materials or were produced through the degradation or redox reactions of macromolecules within them, while others resulted from microbial metabolic activity. Additionally, certain pyrazines, alkanes, and alkenes were detected, all of which contribute to characteristic flavor profiles and enhance the natural perception of yoghurt’s taste.

A comprehensive analysis of four yoghurt samples revealed that OAY contained a slightly lower variety of volatile compounds than AY, yet higher than both OY and OAM. Furthermore, OAY exhibited a total volatile compound content of 523.02 μg/kg, surpassing both OY (397.43 μg/kg) and OAM (195.73 μg/kg). Compared to single-ingredient yoghurts and pre-fermented samples, the mixed-ingredient fermented yoghurt (OAY) exhibited richer and more complex flavor characteristics, rendering it more readily accepted by consumers.

### 3.6. Analysis of Total Phenolic Content and Antioxidant Activity

Figure 6 illustrates the total phenolic content and antioxidant capacity of different samples. Phenolic compounds have garnered significant attention due to their efficacy and safety in treating human diseases such as cancer and cardiovascular disorders [55]. As depicted in Figure 6a, the total phenolic content of the three yoghurt samples containing oat components was significantly higher than that of AY (*p* < 0.05). This may be attributed to the substantial presence of phenolic substances in oats, including phenolic acids, flavonoids, and phytate [56]. In almonds, phenolic compounds are primarily distributed in the pericarp [57]; however, the raw material used in AY during the production process described in Section 2.2 was blanched almonds. Comparing OAY and OAM revealed a significant increase in total phenolic content in the fermented yoghurt samples (*p* < 0.05). This phenomenon arises from the synergistic effects of lactic acid bacteria’s enzymatic action, disruption of cell wall structures, and microbial metabolic transformations. This collaborative process releases phenolic compounds—which were previously bound and difficult to detect—into a free state while partially generating new bioactive constituents. Consequently, the total phenolic content exhibits a marked increase. This has been confirmed during the fermentation of soybean yoghurt [58] and quinoa yoghurt [42].

To investigate the antioxidant activity of yoghurt extracts, various methods including DPPH, ABTS, and hydroxyl radical scavenging activity were employed for evaluation. As shown in Figure 6, the DPPH radical scavenging rate increased with rising polyphenol content, consistent with previous research findings [59]. In contrast, the ABTS and hydroxyl radical scavenging rates did not exhibit the same trend. This discrepancy stems from the differing phenolic compositions of the oat and almond raw materials. Oats primarily contain ferulic acid, whereas almonds are rich in other types of phenolic compounds. These distinct phenolic substances may exhibit variations in their antioxidant mechanisms, thereby leading to differing scavenging effects on ABTS and hydroxyl radicals [13,60,61]. Moreover, during fermentation, microorganisms release bound phenolic compounds through enzymatic action, generating bioactive peptides and organic acids. This process enhances synergistic interactions between oat polysaccharides, almond-derived polyphenols, and vitamin E [56,62]. This process releases multiple products with antioxidant activity, including free amino acids, peptides, and other small-molecule compounds. These products possess hydrogen-donating capacity, enabling effective radical scavenging [63]. Consequently, OAY exhibited significantly higher ABTS and hydroxyl radical scavenging rates compared to the other three sample groups (*p* < 0.05).

### 3.7. Sensory Evaluation Analysis

**Figure 7:**
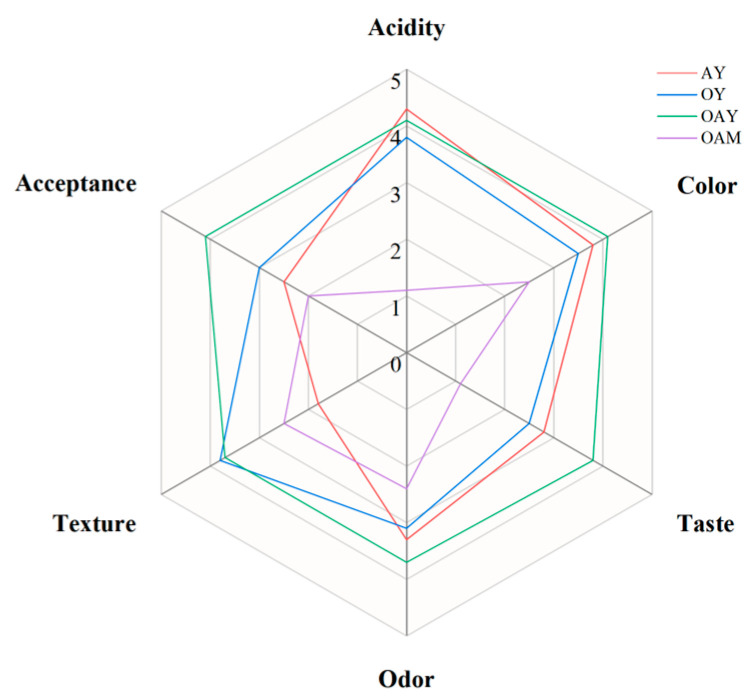
Sensory evaluation scores of different samples.

The sensory attributes of AY, OY, OAY and OAM are presented in Figure 7. Studies have demonstrated that fermentation significantly improves the sensory quality of plant-based products [64]. Fermented samples generally received higher scores than the unfermented OAM. OAM exhibited the lowest acidity, flavor and overall acceptance, which is consistent with its higher pH and the absence of fermentation-derived aroma compounds (Section 3.2). Among fermented samples, OAY showed a well-balanced acidity and achieved the highest scores in taste, odor and overall acceptance. This improvement can be attributed to oat–almond co-fermentation, which enhanced flavor complexity, as supported by the higher abundance of volatile compounds identified by GC–MS (Section 3.5). OY obtained the highest texture score, likely due to the strong water-holding and thickening effects of oat β-glucan, while OAY also demonstrated favorable texture perception, in agreement with its dense multiphase gel structure and rheological properties (Section 3.3 and Section 3.4). Overall, oat–almond co-fermentation effectively improved the sensory quality and acceptability of plant-based yoghurt.

## 4. Conclusions

This study systematically compared OY, AY, OAY and OAM to evaluate the effects of raw material combination and lactic fermentation on the quality of plant-based yoghurt alternatives in terms of physicochemical properties, structural and rheological behavior, volatile flavor profile, antioxidant-related activities, and sensory attributes. The results demonstrated that oat–almond co-fermentation effectively integrated the nutritional and structural characteristics of both raw materials, resulting in a more balanced nutritional composition and significantly improved textural stability, flavor complexity, and overall sensory acceptance compared with single-ingredient yoghurts and the unfermented system. The interaction between oat β-glucan and almond proteins during fermentation was associated with the formation of a dense multiphase gel network, thereby enhancing water-holding capacity, viscosity, and gel strength in the OAY group. Moreover, co-fermentation promoted the generation of volatile flavor compounds, alleviated the inherent raw plant notes of the plant-based matrix, and enhanced antioxidant potential through the release of bound phenolic compounds. Consistently, the sensory evaluation results indicated that OAY exhibited higher overall acceptance than the other samples.

However, there were still some limitations in this study. Only one oat–almond ratio and a single commercial starter culture were evaluated, and storage stability and the bio-accessibility of active compounds during in vitro digestion were not investigated. So, future studies could further explore the application potential of oat–almond fermented plant-based yoghurt substitutes by optimizing formulation ratios, investigating differences in fermentation strains, evaluating shelf life quality changes, and assessing digestive behavior, to provide a more comprehensive assessment.

## Figures and Tables

**Table 1:** Nutritional composition of different samples.

	Nutritional Components (g/100 g)			
	Protein	Fat	Carbohydrates	TSC
AY	3.29 ± 0.02 ^a^	8.89 ± 0.03 ^a^	2.16 ± 0.03 ^c^	5.25 ± 0.03 ^c^
OY	2.39 ± 0.03 ^c^	3.30 ± 0.02 ^c^	9.93 ± 0.04 ^a^	14.35 ± 0.04 ^a^
OAY	2.87 ± 0.02 ^b^	5.18 ± 0.04 ^b^	6.17 ± 0.02 ^b^	11.69 ± 0.02 ^b^
OAM	2.85 ± 0.01 ^b^	5.35 ± 0.03 ^b^	6.52 ± 0.01 ^b^	11.69 ± 0.03 ^b^

Values are expressed as mean ± standard deviation (*n* = 3). Different letters within the same column indicate significant differences (*p* < 0.05); identical letters denote no significant difference (*p* > 0.05).

**Table 2:** Physicochemical and microbiological analysis of different samples.

	pH	TTA (°T)	WHC (%)	Viscosity (mPa·S)	TLC (log10 CFU/g)
AY	4.20 ± 0.02 ^d^	75.32 ± 3.03 ^a^	29.08 ± 0.05 ^d^	2224.30 ± 30.84 ^d^	3.8 ± 0.13 ^c^
OY	4.73 ± 0.05 ^b^	71.76 ± 4.56 ^c^	84.27 ± 0.06 ^a^	6025.26 ± 65.95 ^a^	4.7 ± 0.15 ^b^
OAY	4.49 ± 0.02 ^c^	74.05 ± 4.29 ^b^	58.08 ± 0.05 ^b^	5381.49 ± 42.12 ^b^	7.1 ± 0.06 ^a^
OAM	6.35 ± 0.01 ^a^	23.65 ± 0.05 ^d^	47.32 ± 0.06 ^c^	5086.47 ± 54.77 ^c^	-

Values are expressed as mean ± standard deviation. Different letters within the same column denote significant differences (*p* < 0.05); identical letters indicate no significant difference (*p* > 0.05). “-” indicates not detected.

**Table 3:** Textural characteristics of different samples.

	Hardness (g)	Viscosity (g·s)	Elasticity	Cohesion	Adhesion (g)
AY	10.00 ± 0.01 ^d^	−0.21 ± 0.03 ^a^	0.32 ± 0.01 ^a^	0.21 ± 0.05 ^a^	9.10 ± 0.14 ^d^
OY	55.33 ± 1.15 ^a^	−25.32 ± 9.62 ^c^	0.77 ± 0.04 ^c^	0.74 ± 0.09 ^b^	40.68 ± 4.49 ^a^
OAY	30.67 ± 1.15 ^b^	−16.46 ± 6.91 ^b^	0.85 ± 0.03 ^b^	0.79 ± 0.65 ^b^	24.27 ± 2.57 ^b^
OAM	18.67 ± 1.15 ^c^	−2.44 ± 0.92 ^a^	0.57 ± 0.01 ^d^	0.74 ± 0.01 ^b^	15.74 ± 1.15 ^c^

Values are expressed as mean ± standard deviation. Different letters within the same column denote significant differences (*p* < 0.05); identical letters indicate no significant difference (*p* > 0.05).

**Table 4:** PEN3 electronic nose sensor performance description.

Sensor Serial Number	Sensor Name	Sensor Sensitive Substances
S1	W1C	Aromatic compounds
S2	W5S	Nitrogen oxides
S3	W3C	Ammonia, aromatic compounds
S4	W6S	Hydrogen
S5	W5C	Alkanes, aromatic compounds
S6	W1S	Alkanes
S7	W1W	Sulfides, terpenes
S8	W2S	Alcohols, aldehydes, ketones
S9	W2W	Aromatic compounds, organosulfur compounds
S10	W3S	Long-chain alkanes

## Data Availability

The original contributions presented in this study are included in the article. Further inquiries can be directed to the corresponding author.

## Appendix A. Analysis of Volatile Compounds in Different Yogurt Samples

**Order**	**Compound Name**	**Olfactory Characteristics**	**Content (μg/kg)**			
			**AY**	**OY**	**OAY**	**OAM**
Aldehydes						
1	artificial almond oil	Bitter almond, cherry, nut	29.66	-	1.06	29.91
2	nonadienal	Floral and fruity aroma, oily	69.23	34.4	-	26.8
3	2-Decenal	Intense citrus peel, fat, waxy	60.84	11.78	39.62	6.32
4	2-nonenal	Strong fat, cucumber, and melon-like	60.84	-	30.1	0.53
5	10-undecen-1-ene	A pungent, orange-like aroma	6.31	10	31.54	1.94
6	aldehyde C-9	Rose, citrus	64.65	-	14.13	3.36
7	trans-2-hexenal	Fresh fruits, green leaves, and a refreshing aroma	3.55	-	1.54	1.94
8	anti-2-dodecyl-1-ol	Coriander flavor	0.63	-	-	-
9	capraldehyde	Sweet, citrus, waxy, and floral	1.04	-	2.4	-
10	myristic aldehyde	Iris-like odor	0.43			
11	2-dodecanal	Intense, citrus and orange peel-like aroma	0.48	-	20.96	6.32
12	(Z)-9-tetradecenal	Citrus-like, iris-like, and iris root-like	0.48	-	-	-
13	erythrocitrin	Intense orange, tangerine, and orange-like colors	0.48	-	-	-
14	cis-7-tetradecenal	Citrus-like, iris-like, iris root-like	0.48	-	-	-
15	9-Heptadecenal	Aromatic aroma	0.48	3.31	-	-
16	2,4-Decadienal	Exhibits a strong chicken aroma and chicken oil flavor	0.63	-	-	-
17	anti, anti-2,4-dodecadienal	Oleoresin, fatty and milk aroma, citrus-like	0.48	2.8	1.15	6.32
18	10-18-aldehyde	Wax, citrus, and soap-like odors	7.7	-	-	-
19	hexahydrobenzaldehyde	Aldehyde aroma, green grass aroma	-	3.76	-	-
20	2-Ethylhexanal	Fresh grass and fatty aroma	-	2.87	-	-
21	octanal	Citrus, fatty, and waxy aromas	-	1.27	19.81	-
22	peach aldehyde	Intense peach-like aroma	5.69	-	-	-
23	undecanal	Rose, floral, fruity, and sweet orange	-	-	12.33	2.02
24	5-Octenal	Greenish aroma, fat, citrus-like and melon-like	-	0.76	16.53	0.49
25	3-Methoxypropionaldehyde	Green grass, sweet fragrance	-	-	-	7.37
26	(Z)-2-heptenal	Fat, green grass, lipid-like substance	-	14.58	-	1.62
Acids						
1	arachidonic acid	Fish oil and fatty aroma	4.94	-	-	-
2	cis-18-carb-11-ene	Milkshake	0.44	-	-	-
3	trans-18-carb-13-ene	Lavender milk fragrance	0.44	-	-	-
4	3-hydroxylauryic acid	Fatty odor and soap-like smell	0.66	-	-	-
5	trans-13-eicosapentaenoic acid	lipa	4.50	-	-	-
6	9-Decanoic acid	Fatty acids and waxy aroma, with slight fruity and frankincense notes	-	0.43	2.02	-
7	benzoic acid	Slightly almond-like aroma	-	-	0.96	-
8	octadecenic acid	Lard	-	-	1.06	-
9	3,5-Dimethoxycinnamic acid	Faintly aromatic, sweetly scented	-	1.21	-	0.57
10	cis-5,8,11,14,17-eicosapentaenoic acid	Fish oil flavor	-	-	-	1.38
esters						
1	10-oleoyl ethyl ester	Edible, fruity, and wine aromas	20.31	-	1.25	-
2	phenylethyl acetate	Rose fragrance, honey-like aroma, apple scent, cocoa and whisky-like notes	15.24	13.2	-	-
3	nonyl acetate	Mushroom and gardenia fragrance	5.23	-	1.38	-
4	10-Butyl-14-ene-1-ol acetate	Aromatic fruit flavor	6.96	-	-	-
5	dihydroactinidiolide	Tea aroma, hay aroma, sweet fruit aroma, and a faint mint-like scent	5.69	-	-	-
6	tetradecenolide	Coconut and nuts	5.69	-	-	-
7	ethyl 5-methyl-nonyl phosphate	Fruit, sweet, and apple-like aromas	8.27	-	-	-
8	ethyl caprate	Fruit aroma, sweet aroma, and a brandy-like fragrance	8.27	-	1.23	0.65
9	ethyl 9-oxononyl phosphate	Fruit aroma, waxy notes, and a subtle hint of grass	8.27	-	1.15	0.61
10	tridecylbenzene	Faint waxy fragrance	-	9.11	-	-
11	valeric acid-10-undecenyl ester	Fruity	-	2.68	-	-
12	triethylstearate	Fruit, wax, and brandy-like aromas	-	-	18.75	-
13	octyl phenylacetate	The sweet fragrance of roses and honey, with notes of fruit and animal musk	-	-	18.89	-
14	(Z)-9-Ethyl octadecenoate	The fragrance of flowers	-	-	1.83	-
15	heptyl formate	Pear and apple-like fruit fragrance, floral aroma, iris and rose scents	-	2.54	2.31	-
16	levoepine	A fragrance reminiscent of roses, with undertones of apple and pineapple, accompanied by a sweetness akin to apples	-	-	1.15	-
17	methyl caprylate	Aroma of wine, fruit, and citrus	-	-	1.15	-
18	methyl linolenate	A mild, characteristic aroma of fat and wax	-	-	1.83	-
19	γ-dodecyl lactone	Aroma of cream, peaches, and pears	-	-	1.54	0.32
20	methyl 8-methyl-nonyl ester	The fragrance of fruit	-	-	1.25	-
21	ethyl pelargonate	Fats, fruits, and brandy-like aromas	-	-	1.25	0.85
22	8-hydrocoumarin	Sweet coconut and vanilla-like fragrance	-	-	1.44	-
23	ethyl laurate	Fruity, waxy, fatty	-	0.75	-	0.65
24	tetradecanolide	Musk, peach, cream	-	-	-	0.4
25	2-Methylbutyl formate	Fruit aroma, sweet aroma, similar to rum	-	-	-	17.93
alcohols						
1	1-octen-3-ol	Mushroom, lavender, rose, and hay scents	20.31	2.87	4.81	0.4
2	1-nonen-3-ol	Aromatic odor	20.31	2.84	4.47	-
3	2-Ethylhexanol	Sweet taste with a faint floral aroma	60.84	11.78	-	6.32
4	n-caprylic alcohol	Citrus, orange peel, and rose-like scents	25.31	-	6.25	-
5	3,7-Dimethyl-1-octanol	Sweet rose fragrance with lemongrass and geranium notes, sweet with a spicy undertone	25.31	-	-	-
6	tetrahydrolychol	Lavender-like floral fragrance with a hint of green and spicy notes	11.01	-	-	-
7	2-Propyl-1-heptanol	Fatty and citrus-like aroma	11.01	0.96	2.5	-
8	1-Butanol	Aromatic, fatty, and cucumber-like fragrance	64.65	10.03	14.13	-
9	anti-2-undecenol	Floral fragrance, fruity aroma	65.69	-	2.4	-
10	nonenol	The aroma of wax melon/cucumber	62.1	20.31	1.83	-
11	2-Decene-1-ol	Wax, fresh air, citrus, rose, and yarrow	67.12	-	2.4	-
12	1-Nonyl alcohol	Citrus, rose-like, and fatty aromas	3.55	3.19	-	3.12
13	decenol	Rose, wax, and aldehyde fragrances	0.63	-	-	-
14	1-hexadecanol	Rose fragrance	4.91	-	9.52	-
15	2,5-Pentadecadien-1-ol	The fresh fragrance of cucumber and watermelon rinds	6.66	-	1.25	-
16	cyclohexadecanol	Mild notes of wood, powder, and musk-like aromas	7.74	-	-	-
17	isorosanone	Pine and herbaceous aromas	-	9.11	-	-
18	2-Butyl-1-octanol	Fruit aroma and sweetness	-	1.97	-	-
19	undecyl alcohol	Citrus aroma	-	3.19	-	-
20	dodecene-3-ol	Fatty aroma, citrus scent, waxy odor, and soap-like smell	-	-	4.81	-
21	carveol	The fragrance of lavender and coriander	-	-	1.15	-
22	1-Octen-3-ol	A greenish aroma reminiscent of mushrooms and soil, with notes of herbs, medicinal plants, and violet leaves	-	-	19.81	2.59
23	N-nonadecanol	Sweet and creamy notes with a hint of rose and citrus	-	-	7.79	3.12
24	1-hepten-3-ol	Mushrooms, soil	-	-	-	2.59
25	benzil alcohol	Faint floral and sweet fragrance	-	-	-	2.83
26	phenethyl alcohol	Rose, honey, floral fragrance	-	-	-	1.21
27	isopinocampheol	Pine, camphor, mint	-	-	-	0.36
ketone						
1	5-methyl-1-phenylhex-5-ene-1-one	Fruit aroma or green aroma	15.24	12.11	-	-
2	pyran-4-one	Herbaceous odor similar to hay	39.77	-	3.32	2.92
3	exalton	Musk fragrance	6.35	-	-	-
4	4-methylcyclopentadecanone	Mellow musk, powder, and wood fragrance	6.57	-	2.69	-
5	methyl coumarin	Aromatic notes, coumarin-like sweetness, and nutty undertones	11.22	20.13	-	-
6	ionone	Warm woody notes with a pronounced violet fragrance	-	0.64	-	-
7	6-methyl-3-heptanone	Fruit aroma, grassy aroma	-	2.87	3.56	-
8	6-methyl-5-hepten-2-one	Fruit aroma and lemongrass aroma	-	-	1.06	-
9	3-hydroxy-2-butanone	The aroma of cream, butter, and yoghurt	-	-	18.97	16.89
10	2-nonyl ketone	Fruit aroma, sweet aroma, similar to cheese	-	-	-	2.02
11	cyclopentadecanonum	Sandalwood, Aucklandia	-	-	-	0.81
12	2-(2-octenyl)cyclopentanone	Fruit fragrance, jasmine	-	-	-	0.45
hydrocarbons, alkenes, pyrazines, and other compounds						
1	ethylbenzene	Aromatic odor	37.65	8.34	16.77	4.29
2	o-xylene	Aromatic odor	246.39	50.51	15.52	4.41
3	p-xylene	Aromatic odor	256.81	8.58	15.38	5.32
4	m-xylene	Aromatic odor	38.73	8.54	15.38	4.29
5	2,5-Dimethylpyrazine	The aroma of roasted beans and the scent of chocolate and cream	34.53	-	4.33	-
6	2,6-dimethylpyrazine	The smell of coffee and roasted peanuts	35.32	-	4.32	-
7	2-Ethyl-6-methylpyrazine	Nuts, baked aromas, and roasted potatoes	50.21	1.34	4.23	-
8	2-Ethyl-3-methylpyrazine	Nutty aroma, peanut aroma, grain-like aroma, bread aroma	27.73	-	4.45	-
9	2-ethanyle-3,5-dimethylpyrazine	The aroma of whisky and roasted peanuts	39.77	-	-	-
10	3-Ethyl-2,5-dimethylpyrazine	The roasted cocoa and almonds emit a similar aroma	41.11	-	7.21	-
11	4,6,9-Tetradecatriene	Fatty aroma, green aroma, or an aroma similar to that of cucumber or melon	6.21	-	-	-
12	2-Butylfuran	Herbal and fruit aromas	-	34.77	-	-
13	2-pentylfuran	The aromas of bean, fruit, earth, green, and vegetables	69.23	34.4	-	-
14	terpenoid	Mint-like aroma	-	-	1.06	-
15	caryophyllene oxide	Cinnamomum, pungent, faint camphor odor	-	-	-	0.4
16	cinnamene	Mugwort, pungent, earthy, and faint camphor-like	-	-	1.25	-
17	azulene	Cinnamomum cassia, camphor, faint fragrance	-	-	-	3.12
